# Sex differences in the association of long-term exposure to heat stress on kidney function in a large Taiwanese population study

**DOI:** 10.1038/s41598-024-65741-7

**Published:** 2024-06-25

**Authors:** Yi-Kong Chen, Ping-Hsun Wu, Pei-Yu Wu, Yi-Chun Tsai, Yi-Wen Chiu, Jer-Ming Chang, Chih-Hsing Hung, Chih-Da Wu, Chao-Hung Kuo, Yu-Chee Tseng, Szu-Chia Chen

**Affiliations:** 1 Graduate Institute of Smart Industry and Green Energy, College of Artificial Intelligence, National Yang Ming Chiao Tung University, Tainan, Taiwan; 2 Department of General Medicine, Kaohsiung Medical University Hospital, Kaohsiung Medical University, Kaohsiung, Taiwan; 3 Division of Nephrology, Department of Internal Medicine, Kaohsiung Medical University Hospital, Kaohsiung Medical University, Kaohsiung, Taiwan; 4 Department of Internal Medicine, Kaohsiung Municipal Siaogang Hospital, Kaohsiung Medical University, Kaohsiung, 812 Taiwan; 5 Faculty of Medicine, College of Medicine, Kaohsiung Medical University, Kaohsiung, Taiwan; 6 Research Center for Precision Environmental Medicine, Kaohsiung Medical University, Kaohsiung, Taiwan; 7 Department of Pediatrics, Kaohsiung Medical University Hospital, Kaohsiung Medical University, Kaohsiung, Taiwan; 8 Department of Pediatrics, Kaohsiung Municipal Siaogang Hospital, Kaohsiung Medical University, Kaohsiung, Taiwan; 9 Department of Geomatics, National Cheng Kung University, Tainan, Taiwan; 10 National Institute of Environmental Health Sciences, National Health Research Institutes, Miaoli, Taiwan; 11 Innovation and Development Center of Sustainable Agriculture, National Chung-Hsing University, Taichung, Taiwan; 12 Division of Gastroenterology, Department of Internal Medicine, Kaohsiung Medical University Hospital, Kaohsiung Medical University, Kaohsiung, Taiwan; 13 Department of Computer Science, National Yang Ming Chiao Tung University, Hsinchu, Taiwan

**Keywords:** Wet bulb globe temperature, Kidney function, Sex difference, Taiwan biobank, Climate sciences, Nephrology, Risk factors, Environmental sciences, Outcomes research

## Abstract

The incidence and prevalence of dialysis in Taiwan are high compared to other regions. Consequently, mitigating chronic kidney disease (CKD) and the worsening of kidney function have emerged as critical healthcare priorities in Taiwan. Heat stress is known to be a significant risk factor for CKD and kidney function impairment. However, differences in the impact of heat stress between males and females remains unexplored. We conducted this retrospective cross-sectional analysis using data from the Taiwan Biobank (TWB), incorporating records of the wet bulb globe temperature (WBGT) during midday (11 AM–2 PM) and working hours (8 AM–5 PM) periods based on the participants’ residential address. Average 1-, 3-, and 5-year WBGT values prior to the survey year were calculated and analyzed using a geospatial artificial intelligence-based ensemble mixed spatial model, covering the period from 2010 to 2020. A total of 114,483 participants from the TWB were included in this study, of whom 35.9% were male and 1053 had impaired kidney function (defined as estimated glomerular filtration rate < 60 ml/min/1.73 m^2^). Multivariable analysis revealed that in the male participants, during the midday period, the 1-, 3-, and 5-year average WBGT values per 1 ℃ increase were significantly positively associated with eGFR < 60 ml/min/1.73 m^2^ (odds ratio [OR], 1.096, 95% confidence interval [CI] = 1.002–1.199, *p* = 0.044 for 1 year; OR, 1.093, 95% CI = 1.000–1.196, *p* = 0.005 for 3 years; OR, 1.094, 95% CI = 1.002–1.195, *p* = 0.045 for 5 years). However, significant associations were not found for the working hours period. In the female participants, during the midday period, the 1-, 3-, and 5-year average WBGT values per 1 ℃ increase were significantly negatively associated with eGFR < 60 ml/min/1.73 m^2^ (OR, 0.872, 95% CI = 0.778–0.976, *p* = 0.018 for 1 year; OR, 0.874, 95% CI = 0.780–0.978, *p* = 0.019 for 3 years; OR, 0.875, 95% CI = 0.784–0.977, *p* = 0.018 for 5 years). In addition, during the working hours period, the 1-, 3-, and 5-year average WBGT values per 1 ℃ increase were also significantly negatively associated with eGFR < 60 ml/min/1.73 m^2^ (OR, 0.856, 95% CI = 0.774–0.946, *p* = 0.002 for 1 year; OR, 0.856, 95% CI = 0.774–0.948, *p* = 0.003 for 3 years; OR, 0.853, 95% CI = 0.772–0.943, *p* = 0.002 for 5 years). In conclusion, our results revealed that increased WBGT was associated with impaired kidney function in males, whereas increased WBGT was associated with a protective effect against impaired kidney function in females. Further studies are needed to elucidate the exact mechanisms underlying these sex-specific differences.

## Introduction

Chronic kidney disease (CKD) is characterized by a progressive deterioration in kidney function, defined as an estimated glomerular filtration rate (eGFR) below 60 mL/min/1.73 m^2^, albuminuria, abnormal urine sediment, histology, imaging, renal tubular disorders, or a history of kidney transplantation, and it represents a significant health challenge. It is clinically classified into five stages, from mild impairment (stage 1) to complete renal failure (stage 5)^1^. CKD affects various body systems, and can lead to hypertension, heart failure, and systemic effects such as cardiorenal syndrome and mineral and bone disorders, thereby elevating the risk of all-cause and cardiovascular mortality^2,3^. Moreover, a report from the United States Renal Database System in 2023 highlighted that Taiwan has the highest global incidence and prevalence of dialysis cases^4^. Consequently, mitigating CKD and the worsening of kidney function have emerged as critical healthcare priorities in Taiwan. While diabetes and hypertension are well-known risk factors for CKD, other factors such as environmental exposure, lifestyle, and the use of herbal medicines have also been associated with the disease^5^. Among these risk factors, increasing attention has been placed on environmental exposure, and especially heat stress due to climate change.

Heat stress is defined as a physiological state where the thermoregulatory mechanisms of the body cannot adequately dissipate accumulated heat, leading to an increase in core body temperature^6^. This phenomenon, exacerbated by climate change, has been associated with higher frequencies of heat-related pathologies such as heat cramps, exhaustion, and stroke^7^. Moreover, increases in the frequency, duration, and severity of extreme heat events due to climate change continue to exacerbate the impact on public health^8^. In addition, factors such as elevated daytime temperatures, reduced cooling at night^9^, and increased levels of air pollution during heat events further compound the health risks^8^. Occupational exposure to heat stress is particularly acute among workers in outdoor settings and heated environments^10^.

Researches have linked heat stress to a decline in kidney function^11^. The response of the body to heat stress is complex, involving both protective and damaging reactions. Activation of the renin–angiotensin–aldosterone system (RAAS) and renal sympathetic nerve activity reduce renal blood circulation and oxygen delivery, which can amplify the risk of kidney function impairment^12^. Dehydration intensifies these effects by decreasing blood volume and increasing osmolarity, thus further impairing kidney function^13^. In addition, disruptions to renal metabolic pathways may lead to increases in uric acid, inflammatory responses, and oxidative stress, all of which are associated with deteriorating renal health^14^.

On the other hand, sex-based physiological differences significantly influence the body’s response to environmental stressors, including heat stress. Males and females exhibit distinct hormonal profiles, body compositions, and thermoregulatory mechanisms, resulting in varied susceptibility to heat stress and its subsequent impact on kidney function^15–17^. Recognizing these differences is essential for developing effective prevention and intervention strategies.

However, existing literature on the differential impact of heat stress on kidney function between males and females is relatively sparse, particularly studies with a large sample size, and the outcomes of previous investigations have been heterogeneous, reflecting differences in study methodologies^18–20^. Consequently, our study aims to provide a comprehensive analysis of the association of heat stress on kidney function, explicitly accounting for sex-based differences.

In this study, we utilized data from the extensive TWB, which includes over 120,000 participants. We combined these data with information on wet bulb globe temperature (WBGT), a measure that considers temperature, humidity and solar radiation, from the Central Weather Bureau of Taiwan. Through the application of a machine learning model, we carefully analyzed the WBGT data. The primary objective of this research was to investigate the connection between yearly-averaged WBGT and eGFR, with a particular focus on understanding how long-term exposure of heat stress affects kidney function differently in males and females.

## Materials and methods

### Subject recruitment from the TWB

The TWB, initiated by Taiwan’s Ministry of Health and Welfare, was set up to enhance healthcare services, mitigate chronic diseases, and tackle challenges related to the aging population. This comprehensive resource comprises medical, genetic, and lifestyle information from cancer-free individuals aged between 30 and 70 years, drawn from various communities across Taiwan^21,22^. The study was conducted according to the Declaration of Helsinki, and it was granted approval by the Institutional Review Board of Kaohsiung Medical University Hospital (KMUHIRB-E(I)-20210,058), and the TWB was granted approval by the IRB on Biomedical Science Research, Academia Sinica, Taiwan and the Ethics and Governance Council of the TWB. We used first enrolled data of TWB for further analysis. Informed consent was obtained from all subjects and/or their legal guardian(s) in the TWB before enrollment.

After providing consent for participation in the TWB, data from the participants are acquired through structured in-person interviews, physical examinations, and the collection of blood specimens, including medical history (hypertension and diabetes mellitus), height, weight, body mass index (BMI) (kg/m^2^), age, sex, and smoking and alcohol consumption. Laboratory tests for fasting glucose, uric acid, hemoglobin, triglycerides, total cholesterol, high-density lipoprotein cholesterol (HDL-C), low-density lipoprotein cholesterol (LDL-C), eGFR (calculated using the 2021 Chronic Kidney Disease Epidemiology Collaboration creatinine equation)^23^ are also performed.

In addition, digital blood pressure (BP) readings are acquired in a sitting position following standard protocols, with the average of three consecutive readings being utilized in the analysis. Regular exercise was defined as at least 30 min of physical activity at least three times a week. A smoking history was defined as smoking at least one cigarette per day for a minimum of one year. An alcohol history was defined as consuming any alcoholic beverage more than four times a week for at least one year.

### Study participants

**Figure 1:**
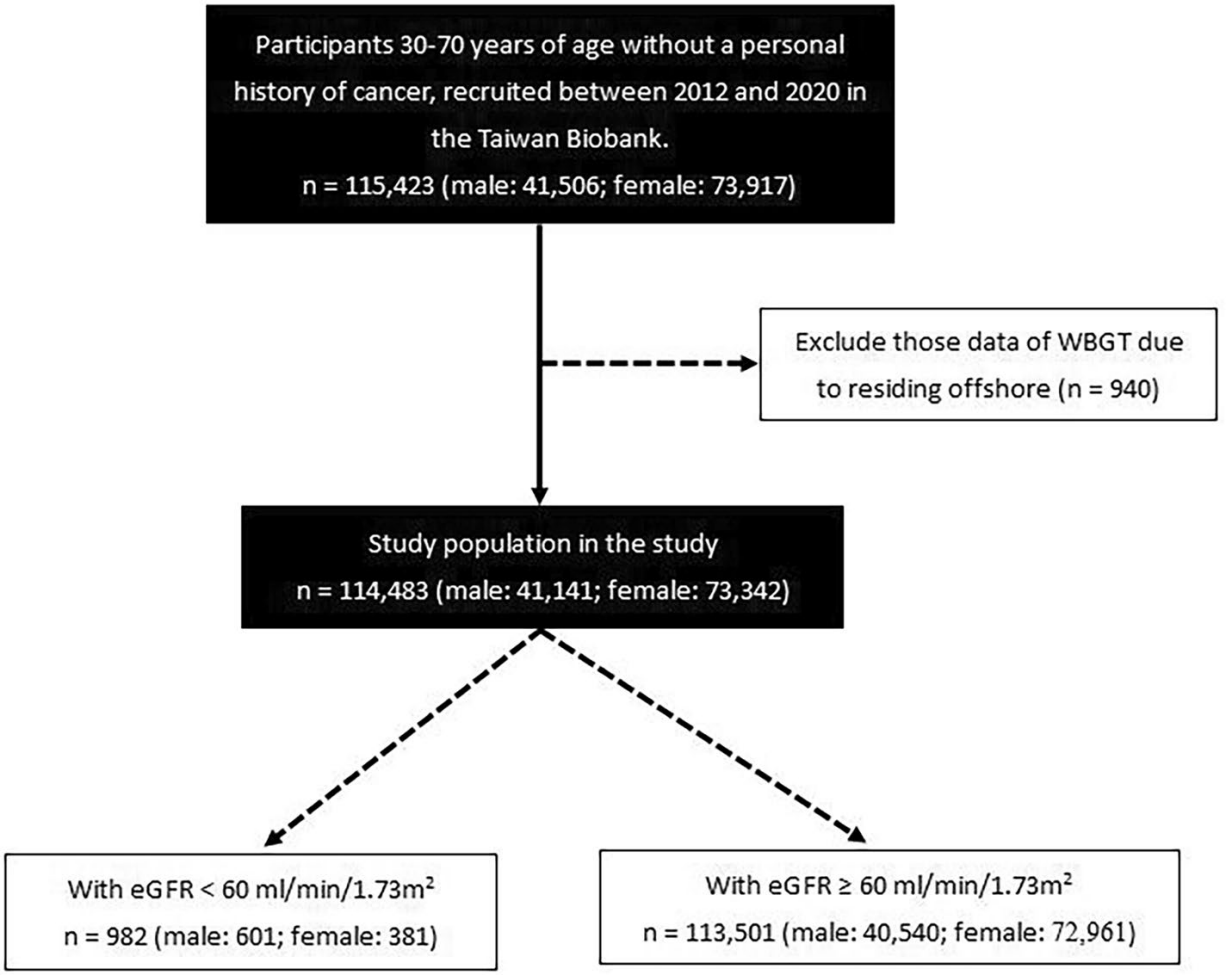
Flowchart of study population.

We identified 115,423 participants from the TWB and excluded those who resided offshore and did not have data on WBGT (n = 940). The remaining 114,483 participants were enrolled (Fig. 1).

### Assessment of WBGT

We obtained hourly temperature data from 453 (427 automatic and 26 manual) weather stations around Taiwan managed by the Central Weather Bureau from 2000 to 2020^24^. The WBGT at each monitoring station was calculated using temperature, humidity, and solar radiation as follows: WBGT = 0.7tnw + 0.2tg + 0.1ta, where tnw represents the natural wet-bulb temperature, tg the globe temperature, and ta the dry bulb temperature. Both tnw and ta are measured across all weather stations, whereas the tg is exclusively measured at the manual stations. In cases where tg measurements were not available at a particular station, the WBGT was estimated utilizing data from the nearest station where tg measurements were recorded^24^. The WBGT values were then categorized into two distinct exposure windows: a working hours period, spanning from 8:00 AM to 5:00 PM, and midday period, between 11:00 AM and 2:00 PM. These hourly WBGT values were then used as the dependent variable in land-use-based spatial machine learning (LBSM) models for predicting high spatial–temporal variations in WBGT in the main island of Taiwan. In addition, factors that could affect WBGT including relative humidity, wind speed, rainfall, and solar declination were also collected. Land use information encompassing parks, water, industrial and residential areas, along with road network details and points of interest such as restaurants and temples was also collected. SHapley Additive exPlanation (SHAP) values were used as a criterion for important predictor selection. A light gradient boosting machine (LightGBM) algorithm coupled with the selected predictor variables was then used to build the prediction model. Incorporating temperature data with other land use/land cover predictor variables significantly enhanced the performance of the LBSM models, achieving an R2 value as high as 0.99^25^. Using these LBSM models, we obtained spatial–temporal WBGT values at a high-resolution grid of 50 m × 50 m.

### Linking data from the TWB and WBGT

Data from the TWB were linked to our WBGT data by residential area at the township level, based on the participants’ residential address, to estimate their heat exposure. Average annual WBGT exposure values were estimated. For each participant, the average WBGT levels for 1, 3, and 5 years preceding the survey year of the TWB were recorded. These WBGT levels were then used as indicators of both short-term and long-term environmental exposure.

### Statistical analysis

Statistical analysis was performed using SPSS 25 for Windows (IBM Inc., Armonk, NY). Data were expressed as percentages or mean ± standard deviation. Differences in categorical variables between groups were analyzed using the chi-square test, and those in continuous variables were analyzed using the independent t-test. Logistic regression analysis was used to identify associations between WBGT with kidney function impairment (defined as eGFR < 60 mL/min/1.73 m^2^). A two-tailed *p* value of less than 0.05 was considered to indicate a statistically significant difference. To investigate the non-linear relationship between WBGT and the risk of eGFR < 60 ml/min/1.73 m^2^, we used restricted cubic splines in SAS (version 9.4, SAS Institute Inc., Cary, NC, USA). The WBGT values were modeled as continuous variables, and the splines were fitted with 4 knots, chosen based on the distribution of WBGT values. The odds ratios (OR) and 95% confidence intervals (CI) were also plotted.

### Ethics declarations

The study was conducted according to the Declaration of Helsinki, and it was granted approval by the Institutional Review Board of Kaohsiung Medical University Hospital (KMUHIRB-E(I)-20210,058), and the TWB was granted approval by the IRB on Biomedical Science Research, Academia Sinica, Taiwan and the Ethics and Governance Council of the TWB. We used first enrolled data of TWB for further analysis. Informed consent was obtained from all subjects and/or their legal guardian(s) in the TWB before enrollment.

## Results

### Clinical characteristics of the participants stratified by sex and eGFR

The clinical characteristics of the study participants stratified by sex and eGFR (< 60 or ≥ 60 mL/min/1.73 m^2^) are shown in Table 1.

**Table 1:** Clinical characteristics of the study participants classified by the presence of different sex and eGFR.

Characteristics	Male (*n* = 41,141)			Female (*n* = 73,342)		
	eGFR < 60 (*n* = 601)	eGFR ≥ 60 (*n* = 40,540)	*p*	eGFR < 60 (*n* = 381)	eGFR ≥ 60 (*n* = 72,961)	*p*
Age (year)	60.6 ± 7.9	49.8 ± 11.4	< 0.001	60.0 ± 7.6	49.9 ± 10.7	< 0.001
DM (%)	42.3	11.8	< 0.001	34.9	7.9	< 0.001
Hypertension (%)	71.9	33.8	< 0.001	65.4	19.3	< 0.001
Smoking history (%)	64.4	57.5	< 0.001	29.0	10.6	0.033
Alcohol history (%)	24.5	18.6	< 0.001	2.1	2.9	0.364
Regular exercise habits (%)	53.1	42.2	< 0.001	53.0	39.5	< 0.001
Systolic BP (mmHg)	135.8 ± 20.2	126.6 ± 17.2	< 0.001	135.0 ± 22.2	117.4 ± 18.5	< 0.001
Diastolic BP (mmHg)	79.4 ± 12.6	78.6 ± 11.0	< 0.001	77.0 ± 12.8	71.4 ± 10.7	< 0.001
BMI (kg/m^2^)	26.1 ± 3.6	25.4 ± 3.6	< 0.001	25.5 ± 4.4	23.6 ± 3.8	< 0.001
Laboratory parameters						
Fasting glucose (mg/dL)	113.1 ± 42.2	99.1 ± 22.8	< 0.001	108.9 ± 38.7	93.9 ± 18.6	< 0.001
Hemoglobin (g/dL)	13.7 ± 1.9	15.1 ± 1.2	< 0.001	12.1 ± 1.7	13.0 ± 1.3	< 0.001
Triglyceride (mg/dL)	169.4 ± 120.8	137.1 ± 118.0	< 0.001	155.4 ± 110.3	102.7 ± 74.0	< 0.001
Total cholesterol (mg/dL)	182.9 ± 39.0	192.1 ± 35.1	< 0.001	205.9 ± 43.7	198.1 ± 36.0	< 0.001
HDL-C (mg/dL)	43.9 ± 11.7	48.0 ± 11.1	< 0.001	52.3 ± 13.0	58.2 ± 13.3	< 0.001
LDL-C (mg/dL)	108.1 ± 32.9	121.9 ± 31.4	< 0.001	121.6 ± 35.9	120.6 ± 31.9	0.529
eGFR (mL/min/1.73 m^2^)	44.6 ± 15.4	101.6 ± 13.3	< 0.001	42.1 ± 16.3	108.4 ± 11.4	< 0.001
Uric acid (mg/dL)	7.2 ± 2.0	6.4 ± 1.3	< 0.001	6.9 ± 1.7	4.8 ± 1.1	< 0.001
WBGT during noon period (11:00 am to 14:00 pm)						
1-year average (per 1 ℃)	27.3 ± 1.1(19.7–29.5)	27.2 ± 1.1(19.4–30.0)	0.541	27.1 ± 1.1(24.0–29.3)	27.3 ± 1.1(19.4–30.0)	0.007
3-year average (per 1 ℃)	27.1 ± 1.1(19.9–29.2)	27.1 ± 1.1(19.1–29.7)	0.686	27.0 ± 1.1(23.6–29.5)	27.1 ± 1.1(19.1–29.7)	0.005
5-year average (per 1 ℃)	27.0 ± 1.1(19.7–29.0)	27.0 ± 1.1(19.1–29.6)	0.598	26.8 ± 1.2(23.4–29.4)	27.0 ± 1.1(19.1–29.6)	0.005
WBGT during work period (8:00 am to 17:00 pm)						
1-year average (per 1 ℃)	25.0 ± 1.2(16.9–27.3)	25.0 ± 1.2(16.4–27.5)	0.580	24.8 ± 1.2(21.8–27.0)	25.1 ± 1.2 (16.4–27.6)	< 0.001
3-year average (per 1 ℃)	24.9 ± 1.2(17.1–27.1)	24.9 ± 1.2(16.4–27.3)	0.505	24.7 ± 1.2(21.9–26.9)	25.0 ± 1.2(16.4–27.3)	< 0.001
5-year average (per 1 ℃)	24.9 ± 1.2(16.8–26.9)	24.8 ± 1.2(16.4–27.2)	0.285	24.6 ± 1.3(21.6–26.7)	24.9 ± 1.2(16.4–27.2)	< 0.001

*DM* diabetes mellitus, *BP* blood pressure, *BMI* body mass index, *HDL-C *high-density lipoprotein cholesterol, *LDL-C* low-density lipoprotein cholesterol, *eGFR* estimated glomerular filtration rate, *WBGT* wet-bulb globe temperature.

WBGT values present as Mean ± Standard Deviation (Minimum—Maximum).

In the male participants, those with an eGFR ≥ 60 mL/min/1.73 m^2^ were older, and had a higher prevalence of diabetes mellitus and hypertension, smoking and alcohol consumption, regular exercise habits, systolic and diastolic BPs, BMI, fasting glucose, triglycerides, eGFR and uric acid levels, and lower hemoglobin, total cholesterol, HDL-C, and LDL-C compared to those with an eGFR < 60 mL/min/1.73 m^2^. Regarding WGBT, there were no significant differences between the two groups in either the midday or working hour periods.

In the female participants, those with an eGFR ≥ 60 mL/min/1.73 m^2^ were older, and had a higher prevalence of diabetes mellitus and hypertension, smoking, regular exercise habits, systolic and diastolic BPs, BMI, fasting glucose, triglycerides, total cholesterol, LDL-C, eGFR and uric acid, and lower hemoglobin, and HDL-C compared to those with an eGFR < 60 mL/min/1.73 m^2^. Regarding WGBT, the participants with an eGFR < 60 ml/min/1.73 m^2^ had significantly higher WGBT values at 1 year, 3 years and 5 years in both midday and working hour periods compared to those with an eGFR ≥ 60 mL/min/1.73 m^2^.

### Association of heat stress with eGFR < 60 ml/min/1.73 m^2^ by sex

Table 2 shows the association between heat stress and eGFR < 60 ml/min/1.73 m^2^ stratified by sex. Multivariable logistic regression was performed adjusting for age, diabetes, hypertension, smoking and alcohol history, regular exercise, systolic and diastolic BPs, BMI, fasting glucose, triglycerides, HDL-C, LDL-C, hemoglobin, and uric acid. The results showed that in the male participants, during the midday period, the 1-, 3-, and 5-year average WBGT values per 1 ℃ increase were significantly positively associated with eGFR < 60 ml/min/1.73 m^2^ (odds ratio [OR], 1.096, 95% CI = 1.002–1.199, *p* = 0.044 for 1 year; OR, 1.093, 95% CI = 1.000–1.196, *p* = 0.005 for 3 years; OR, 1.094, 95% CI = 1.002–1.195, *p* = 0.045 for 5 years). However, no significant associations were found during the working hours period.

**Table 2:** Association of long-term exposure of heat stress with eGFR < 60 ml/min/1.73 m^2^ in different sex using multivariable logistic regression analysis.

WBGT	Male (*n* = 43,603)			Female (*n* = 77,714)		
	Multivariable			Multivariable		
	OR	95% CI	*p*	OR	95% CI	*p*
WBGT during noon period (11:00 am to 2:00 pm)						
1-year average (per 1 ℃)	1.096	1.002–1.199	0.044	0.872	0.778–0.976	0.018
3-year average (per 1 ℃)	1.093	1.000–1.196	0.05	0.874	0.780–0.978	0.019
5-year average (per 1 ℃)	1.094	1.002–1.195	0.045	0.875	0.784–0.977	0.018
WBGT during work period (8:00 am to 5:00 pm)						
1-year average (per 1 ℃)	1.072	0.988–1.164	0.095	0.856	0.774–0.946	0.002
3-year average (per 1 ℃)	1.076	0.990–1.169	0.085	0.856	0.774–0.948	0.003
5-year average (per 1 ℃)	1.078	0.992–1.171	0.077	0.853	0.772–0.943	0.002

Values expressed as odds ratio (OR) and 95% confidence interval (CI).

Adjusted for age, diabetes, hypertension, smoking and alcohol history, regular exercise, systolic and diastolic BPs, BMI, fasting glucose, triglycerides, HDL-C, LDL-C, hemoglobin, and uric acid.

**Figure 2:**
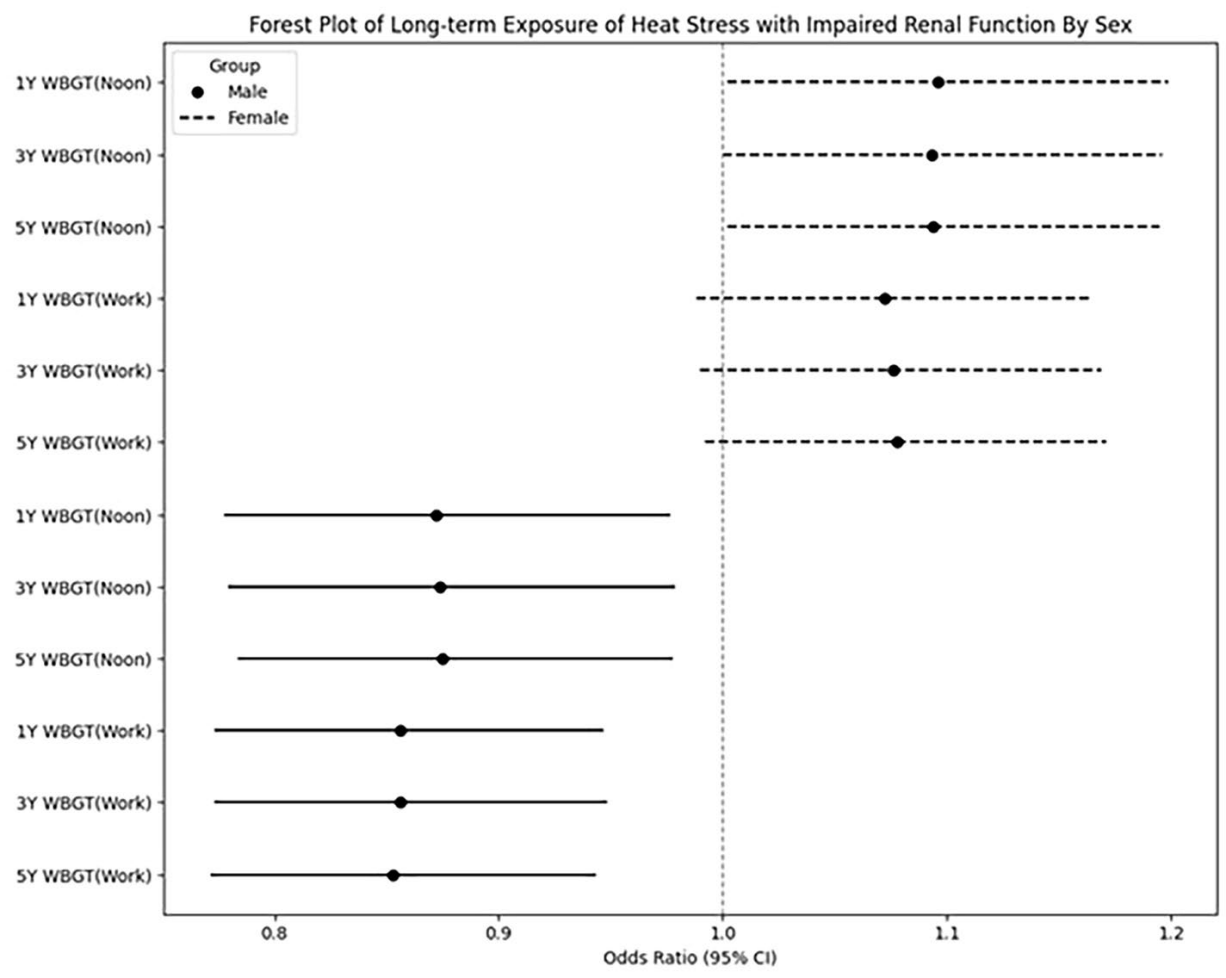
Forest plot of long-term exposure of heat stress with impaired renal function in different sex.

In the female participants, during the midday period, the 1-, 3-, and 5-year average WBGT values per 1 ℃ increase were significantly negatively associated with eGFR < 60 ml/min/1.73 m^2^ (OR, 0.872, 95% CI = 0.778–0.976, *p* = 0.018 for 1 year; OR, 0.874, 95% CI = 0.780–0.978, *p* = 0.019 for 3 years; OR, 0.875, 95% CI = 0.784–0.977, *p* = 0.018 for 5 years). In addition, during the working hours period, the 1-, 3-, and 5-year average WBGT values per 1 ℃ increase were also significantly negatively associated with eGFR < 60 ml/min/1.73 m^2^ (OR, 0.856, 95% CI = 0.774–0.946, *p* = 0.002 for 1 year; OR, 0.856, 95% CI = 0.774–0.948, *p* = 0.003 for 3 years; OR, 0.853, 95% CI = 0.772–0.943, *p* = 0.002 for 5 years). The results in Table 2 are further illustrated in the forest plot presented in Fig. 2.

Supplementary Figs. 1–1 to 2–6 demonstrate the non-linear relationship between WBGT values and eGFR < 60 ml/min/1.73 m^2^ using restricted cubic splines and show the histogram of WBGT value distribution. In the restricted cubic spline plots, the solid line represents the estimated odds ratio for eGFR < 60 ml/min/1.73 m^2^, with dashed lines indicating the 95% confidence intervals.

For male participants (Supplementary Figs. 1–1 to 1–6), higher 1-, 3-, and 5-year average WBGT values are associated with an increased risk of eGFR < 60 ml/min/1.73 m^2^, while lower WBGT levels are linked to a decreased risk during both the midday and work periods. Conversely, for female participants (Supplementary Figs. 2–1 to 2–6), higher 1-, 3-, and 5-year average WBGT values are associated with a decreased risk of eGFR < 60 ml/min/1.73 m^2^, whereas lower WBGT levels show an increased risk during both the midday and work periods.

Multivariable linear regression, adjusted for the same variables, was conducted to assess the relationship between WBGT and eGFR values. In male participants, WBGT was negatively associated with eGFR during both the midday period (β coefficient: − 0.275, 95% CI − 0.377 to − 0.173, p < 0.05 for 1 year; β coefficient: − 0.333, 95% CI − 0.433 to − 0.233, p < 0.05 for 3 years; β coefficient: − 0.291, 95% CI: − 0.402 to − 0.181, p < 0.05 for 5 years) and the working hours period (β coefficient: -0.159, 95% CI − 0.254 to − 0.065, p < 0.05 for 1 year; β coefficient: − 0.226, 95% CI -0.320 to − 0.131, p < 0.05 for 3 years; β coefficient: − 0.158, 95% CI − 0.262 to − 0.054, p < 0.05 for 5 years). For female participants, WBGT was negatively associated with eGFR during the midday period (β coefficient: − 0.065, 95% CI − 0.121 to − 0.008, p < 0.05 for 1 year; β coefficient: − 0.116, 95% CI − 0.171 to − 0.060, p < 0.05 for 3 years; β coefficient: − 0.078, 95% CI − 0.139 to − 0.017, p < 0.05 for 5 years), but no significant associations were found during the working hours period.

## Discussion

In this study, we enrolled 114,483 participants from the TWB and used a machine learning model to determine long-term heat exposure according to the yearly-averaged WBGT during both midday and working hours periods. This determination was based on the residential addresses of the participants. Following stratification by sex, our analysis revealed a significant association between higher WBGT and an increased risk of eGFR < 60 ml/min/1.73 m^2^ during the midday period in male participants, indicating that increased WBGT is linked to a higher risk of impaired renal function in males. Conversely, in female participants, higher WBGT was associated with a decreased risk of eGFR < 60 ml/min/1.73 m^2^ during both midday and working hours, suggesting that elevated WBGT reduces the risk of impaired renal function in females. In the non-linear relationship analysis using restricted cubic splines, supportive results were observed. For male participants, higher 1-, 3-, and 5-year average WBGT values were associated with an increased risk of eGFR < 60 ml/min/1.73 m^2^, while lower WBGT values showed a decreased risk during both midday and work periods. In contrast, for female participants, higher WBGT values were linked to a decreased risk of eGFR < 60 ml/min/1.73 m^2^, whereas lower WBGT values indicated an increased risk in both time periods. These findings highlight the need for further investigation into the sex-specific impacts of WBGT on kidney function.

Heat stress has been associated with compromised kidney function^11^, a concern of particular significance in tropical regions such as Taiwan due to high temperatures and extreme weather conditions. The potential mechanisms linking heat stress to kidney function impairment have been extensively investigated, among which dehydration and hyperthermia are the most prominent^14^. Dehydration has been shown to escalate the risk of acute kidney injury (AKI) under hyperthermic conditions^11^, with this risk positively correlated with the severity of dehydration and hyperthermia^26^. In addition, reactivation of the RAAS and renal sympathetic nervous activity due to dehydration and hyperthermia has been demonstrated to further diminish renal blood flow^27^, potentially impairing oxygen delivery to cortical regions^28^, particularly when activation of the sodium–potassium pump increases oxygen demand (essential for fluid retention). This may then lead to compromised tissue oxygenation, thereby limiting adenosine triphosphate production, which is crucial for normal cellular function^11^. Furthermore, other contributory mechanisms include the amplification of potential pathophysiological pathways ultimately leading to adenosine triphosphate depletion under inflammatory conditions^29^. Indeed, heat-induced exercise is characterized by a pro-inflammatory state, wherein the intensity of inflammation escalates with increasing core body temperature, thereby potentially increasing the risk of AKI^30^. In addition, activation of the fructokinase system has been shown to further exacerbate these pathways^31^. Taken together, this evidence underscores the potential harmful effects of heat stress on kidney function in the general population.

In this study, we identified a significant association between heat stress and an increased incidence of kidney function impairment during the midday period among the male participants. Conversely, a significant negative association was found between long-term heat exposure and the incidence of impaired kidney function during both the midday and working hours periods among the female participants. In a similar investigation, Kim et al.^18^ examined 1,255,671 hospital admissions through the emergency department due to genitourinary diseases between 2003 and 2013 across 16 districts in South Korea, and identified a significant association between high ambient temperature and AKI among men aged over 65 years. However, no significant association was observed among women. The authors attributed this discrepancy to a higher level of exposure to high temperatures among men compared to women. Tawatsupa et al.^19^ investigated the association between occupational heat stress and kidney disease among 37,816 workers in the Thai Cohort Study. Their results showed a greater likelihood of developing kidney disease among men exposed to prolonged heat stress, and a converse trend among women, albeit without statistical significance. Furthermore, they found that exposure to heat stress was more prevalent among men than women, and concluded that this contributed to the difference in the incidence of kidney disease between them. In addition, Schanz et al.^32^ reported that during heat waves, urinary levels of tissue inhibitor of metalloproteinases-2 and insulin-like growth factor binding protein-7, markers of renal tubular stress, were elevated to a greater extent in men compared to women. This finding suggests the presence of sex-specific differences in renal heat tolerance. Previous research has suggested that women may experience lower levels of heat stress than men, even within the same occupation^19^. Furthermore, the impact on kidney function may be more sensitive in men than in women. These observations may clarify the findings of our study.

In addition, while the association between kidney function impairment and heat stress during working hours was not statistically significant, the higher temperatures during the midday period likely had a more substantial impact. This suggests that men’s kidney function may be more affected by the intense heat experienced during midday.

Our study revealed a notable sex difference in the impact of high Wet Bulb Globe Temperature (WBGT) on kidney function. High WBGT was associated with impaired kidney function in males, whereas in females, it was not only unassociated with impairment but also exhibited a protective effect. This disparity can be attributed to the protective role of estrogen and its multifaceted mechanisms in renal protection.

Estrogen, particularly 17β-estradiol (E2), is known for its anti-apoptotic, anti-inflammatory, and vasodilatory effects. These properties provide significant protective benefits to females, particularly in conditions that pose stress to the kidneys. Estrogen’s ability to modulate various cellular pathways, such as TGF-βRI expression, modulation of Th17/Treg cell balance and Cx43 expression which further reduce renal ischemia–reperfusion injury contributes to its protective role^15–17^, which is more pronounced in females due to higher circulating levels of E2 compared to males. Also, during high temperature or heat stress conditions, females may receive enhanced renal protection due to the synergistic effects of E2 and heat shock protein (HSP) induction^33^. HSPs can inhibit the harmful effects of NFκB, a transcription factor caused cellular damage by involving in cellular inflammation and apoptosis^34^. While, heat shock factor 1 activation leads to the increased expression of HSPs during heat stress, estrogen also induces the expression of HSPs^33,35^. In conclusion, the protective role of estrogen, through its regulation of TGF-βRI, modulation of Th17/Treg balance, and induction of HSPs, may contribute to the observed sex differences in renal protection under high WBGT conditions.

Our study found that, in female participants, higher WBGT was associated with a decreased risk of eGFR < 60 ml/min/1.73 m^2^ during both midday and working hours. However, linear regression results indicated a negative association between WBGT and eGFR during the midday period. This discrepancy may arise from the retrospective nature of our study, which relies on existing records and might be subject to biases and unmeasured confounders. Additionally, using eGFR < 60 ml/min/1.73 m^2^ as a cutoff has clinical significance, as patients with eGFR < 60 are at higher risk of progression to end-stage renal disease and require more intensive monitoring and intervention by nephrologists. Further studies are needed to evaluate these effects comprehensively.

This study has several strengths. First, the integration of data from the TWB with LBSM models along with the participants’ residential addresses, which allowed us to individualize the WBGT levels at a high resolution. Second, we used WBGT as a metric for heat exposure, diverging from the conventional reliance on temperature alone. As this approach incorporates humidity, radiation, and wind speed, it allows for a more comprehensive evaluation of environmental heat, thereby augmenting the precision of our study. Third, the inclusion of a substantial cohort from the TWB potentially increased the statistical power of our findings. Consequently, our research contributes to a nuanced understanding of the potential health impacts associated with elevated temperatures. Nonetheless, certain limitations should also be noted. First, the cross-sectional retrospective cohort design of this study inherently involves evidence limitations compared to prospective or randomized controlled trials. Second, our study cohort were relatively healthy, potentially introducing bias regarding the study results due to a lower percentage of kidney function impairment compared to the general population. Lastly, the residential addresses of the participants, while providing insights into their living locations, do not encompass information regarding their workplaces. This lack of data on the participants’ occupational settings may have introduced bias into the estimation of heat stress exposure.

## Conclusions

Our study identified sex differences in the association of long-term heat exposure on kidney function within a substantial Taiwanese cohort. Increased WBGT was associated with impaired kidney function in males, while it was associated with a protective effect against impaired kidney function in females. This analysis linked WBGT data, obtained using a machine learning-based model, to the Taiwan Weather Bureau database by residential area. Further investigations using more broadly representative data or studies designed with a higher level of evidence, such as prospective or randomized controlled trials are warranted to thoroughly explore and validate the impact of heat stress on kidney function in both males and females.

### Supplementary Information

Supplementary Figures.

## Data Availability

The data underlying this study are from the Taiwan Biobank and Taiwan Air Quality Monitoring Database. Due to restrictions placed on the data by the Personal Information Protection Act of Taiwan, the minimal data set cannot be made publicly available. Data may be available upon request to interested researchers. Please send data requests to: S.-C.C., PhD, MD. Division of Nephrology, Department of Internal Medicine, Kaohsiung Medical University Hospital, Kaohsiung Medical University.
