# Supplementary material for: Sex differences in the association of long-term exposure to heat stress on kidney function in a large Taiwanese population study
Source: Sci Rep. 2024 Jun 25;14:14599. doi: 10.1038/s41598-024-65741-7 (PMC11199656; doi:10.1038/s41598-024-65741-7)
Supplement: Supplementary file 1 — Supplementary Figures. [file 41598_2024_65741_MOESM1_ESM.pdf]

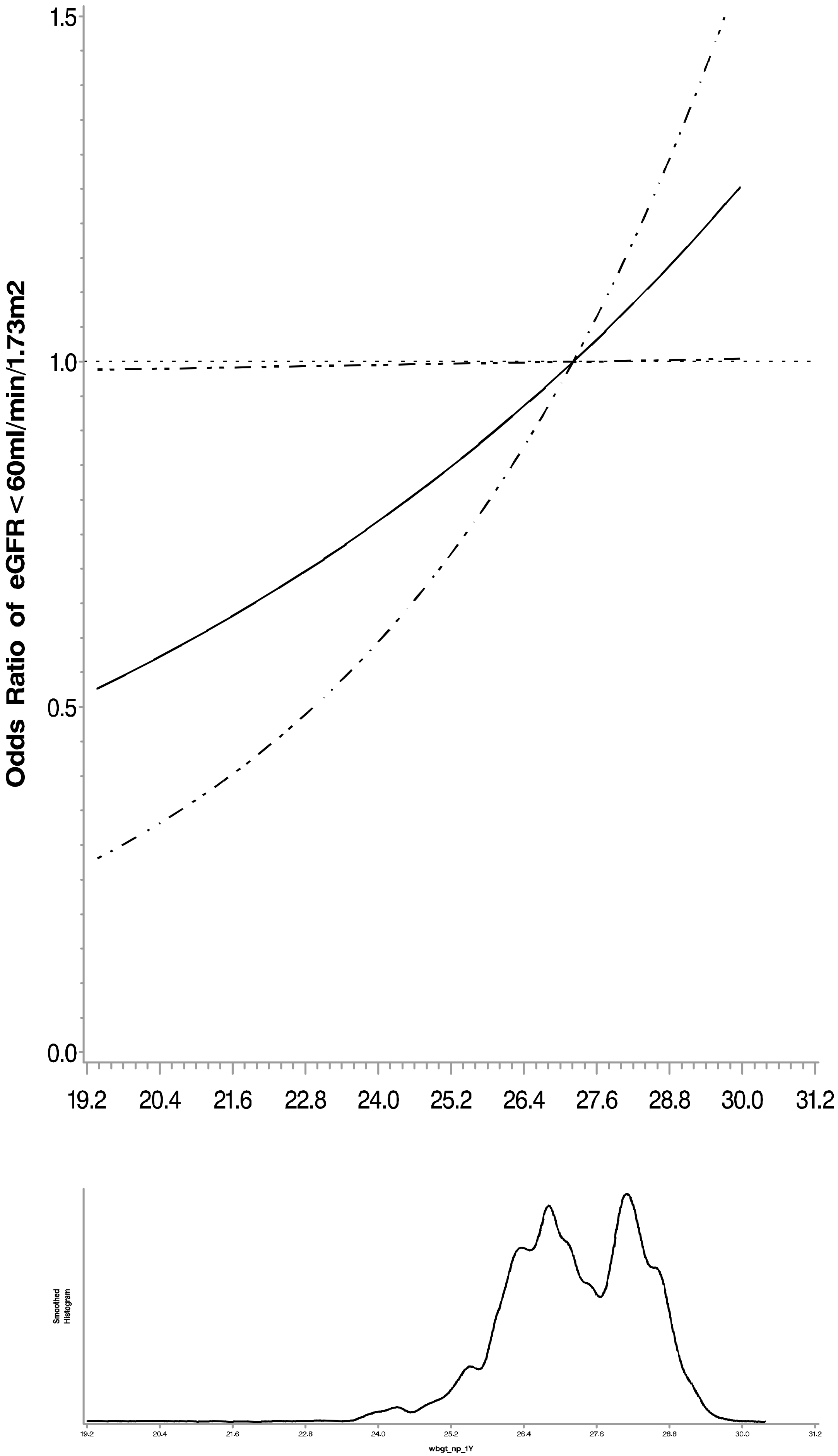

Supplementary Figure 1−1. Restricted cubic spline model of the odds ratios of eGFR<60ml/min/1.73m2 with 1Y WBGT(Noon) (Male)

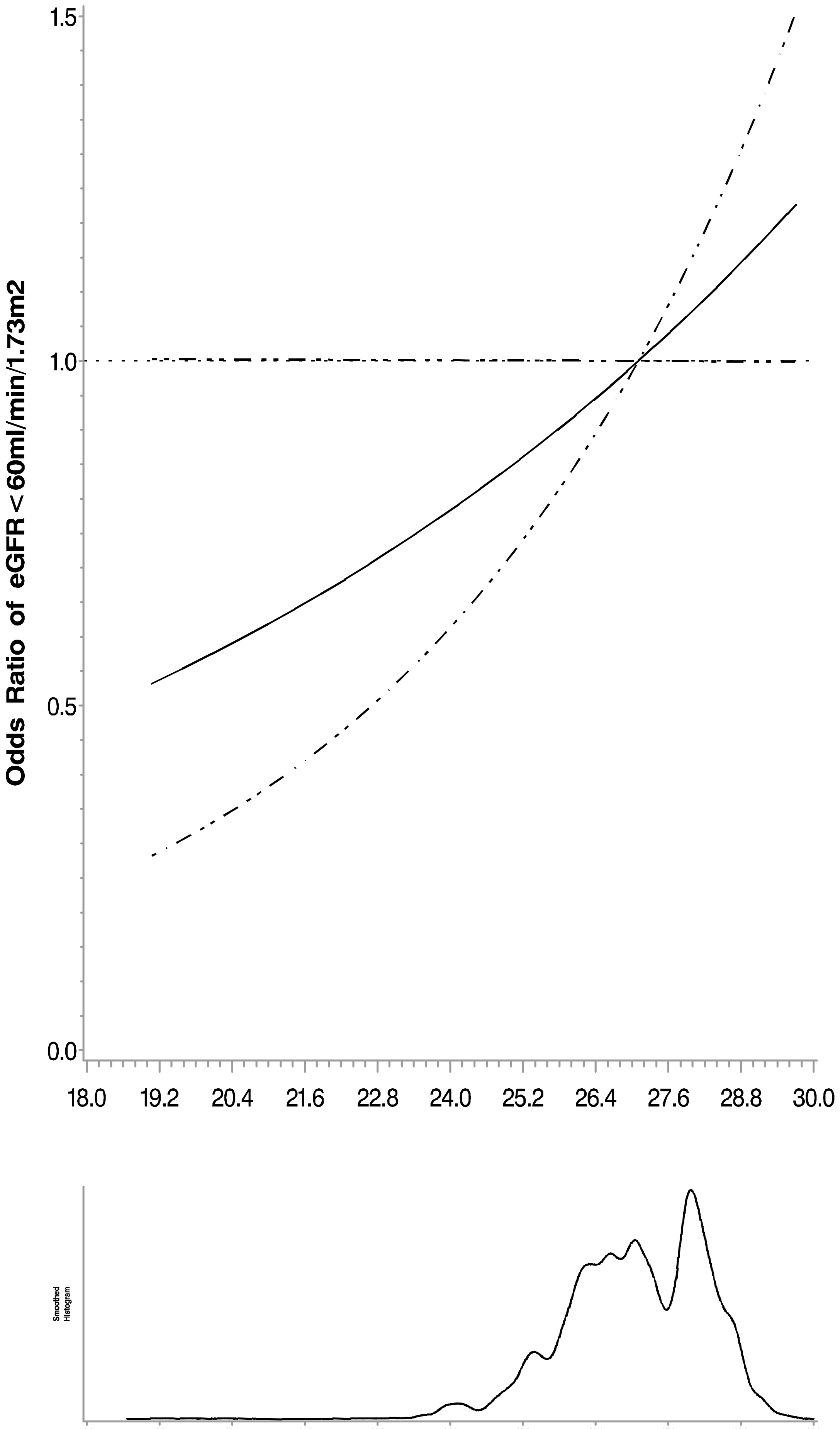

Supplementary Figure 1−2. Restricted cubic spline model of the odds ratios of eGFR<60ml/min/1.73m2 with 3Y WBGT(Noon) (Male)

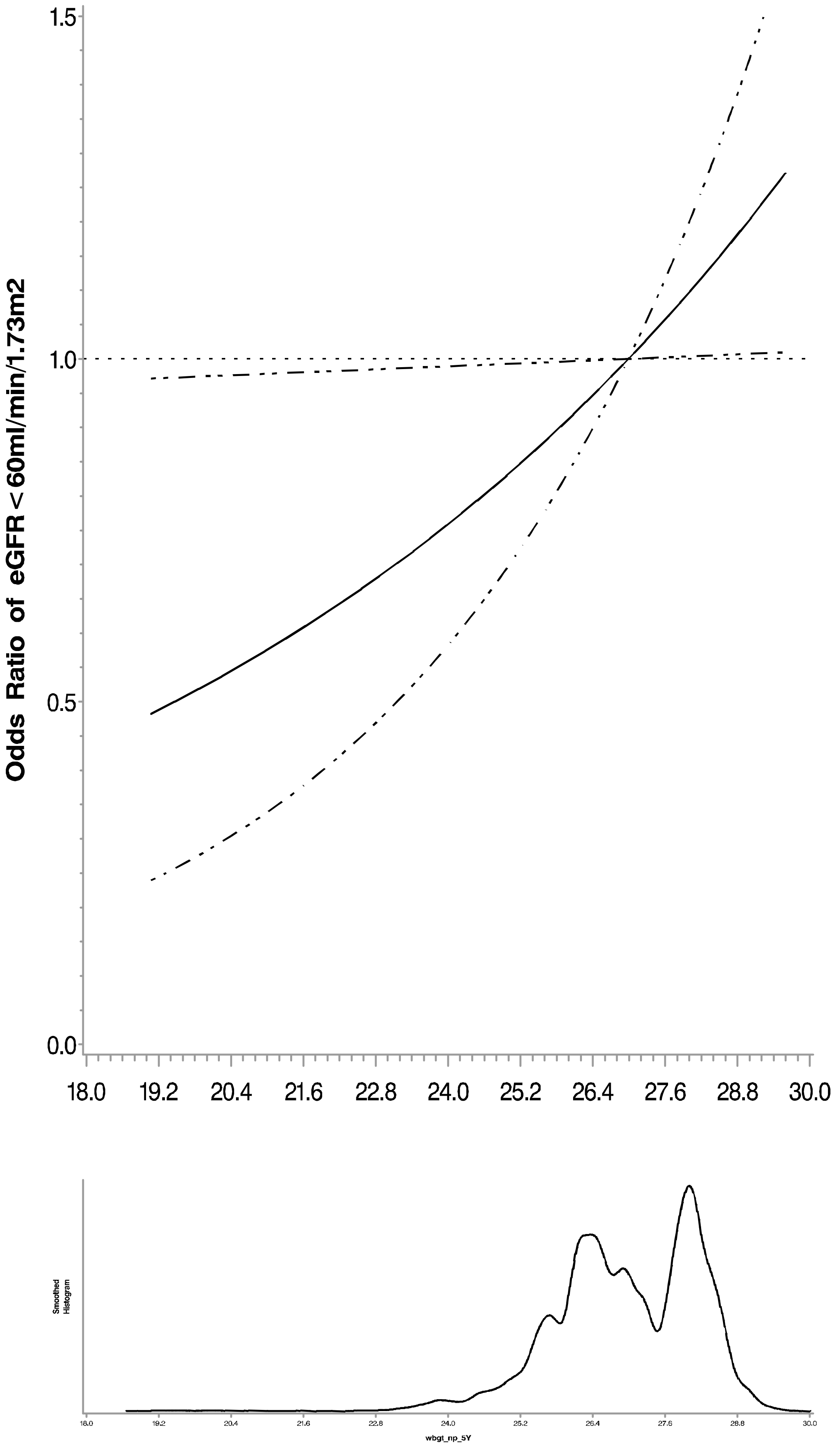

Supplementary Figure 1-3. Restricted cubic spline model of the odds ratios of eGFR<60ml/min/1.73m2 with 5Y WBGT(Noon) (Male)

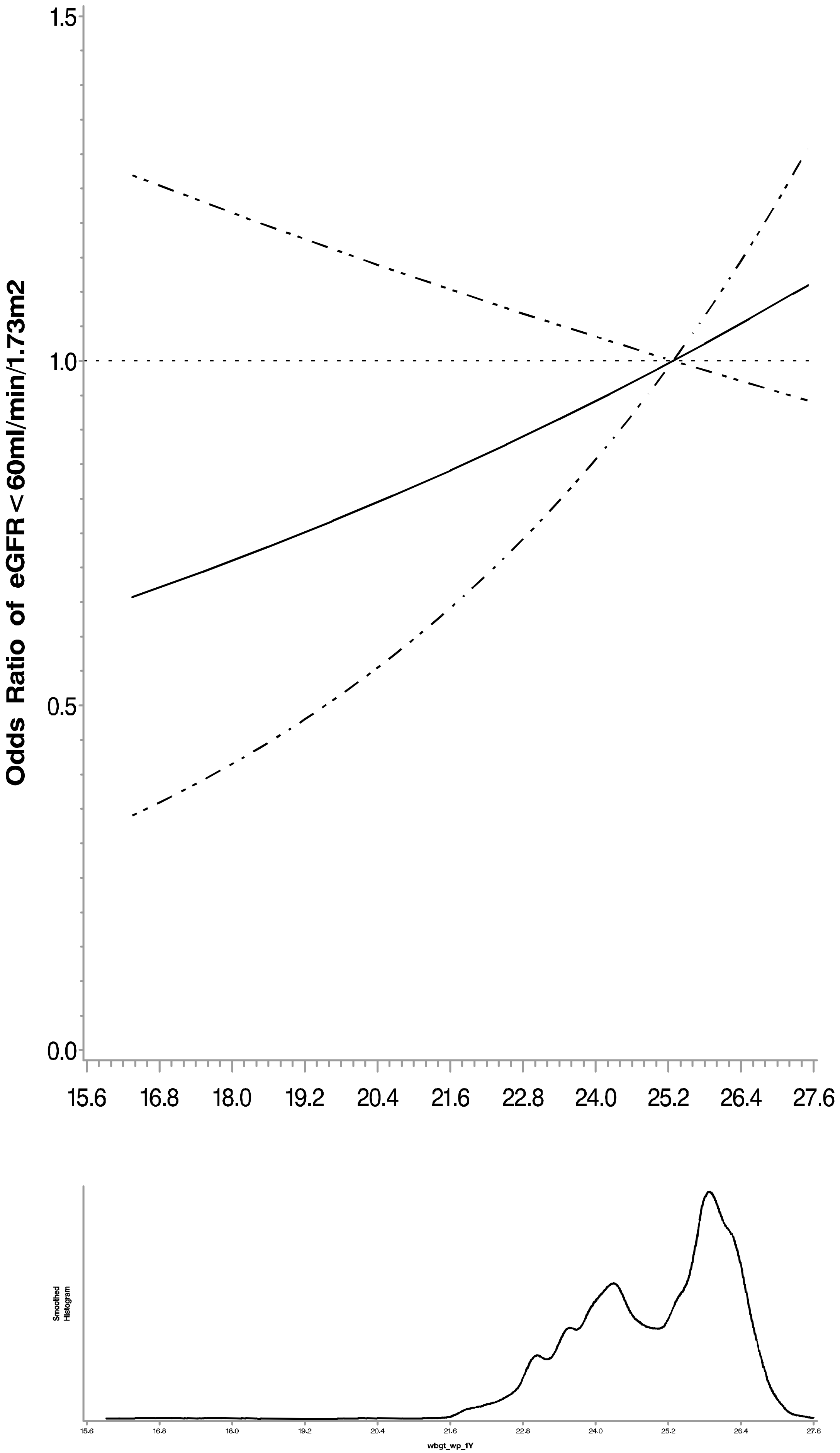

Supplementary Figure 1−4. Restricted cubic spline model of the odds ratios of eGFR < 60ml/min/1.73m2 with 1Y WBGT(Work)(Male)

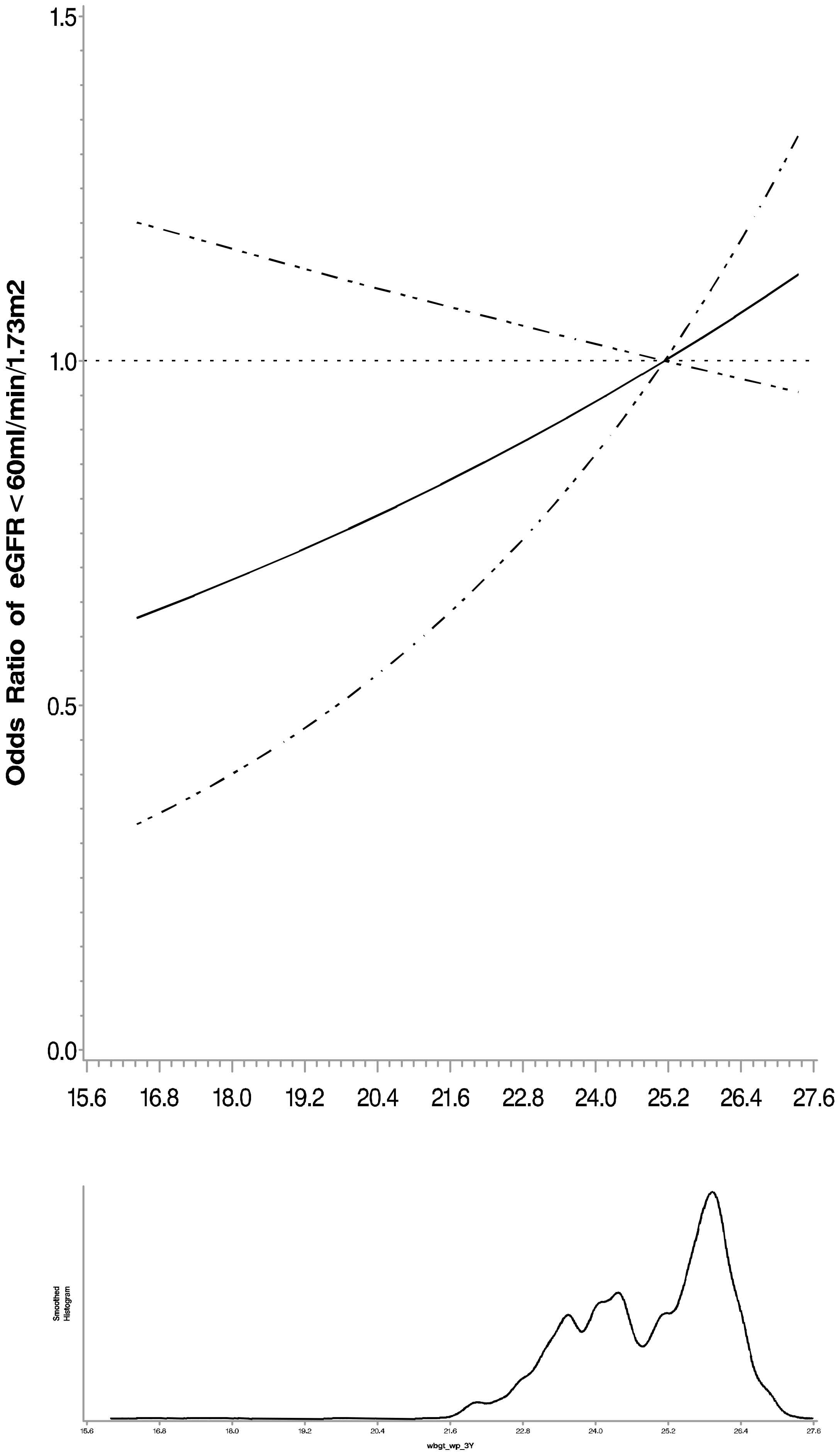

Supplementary Figure 1−5. Restricted cubic spline model of the odds ratios of eGFR<60ml/min/1.73m2 with 3Y WBGT(Work) (Male)

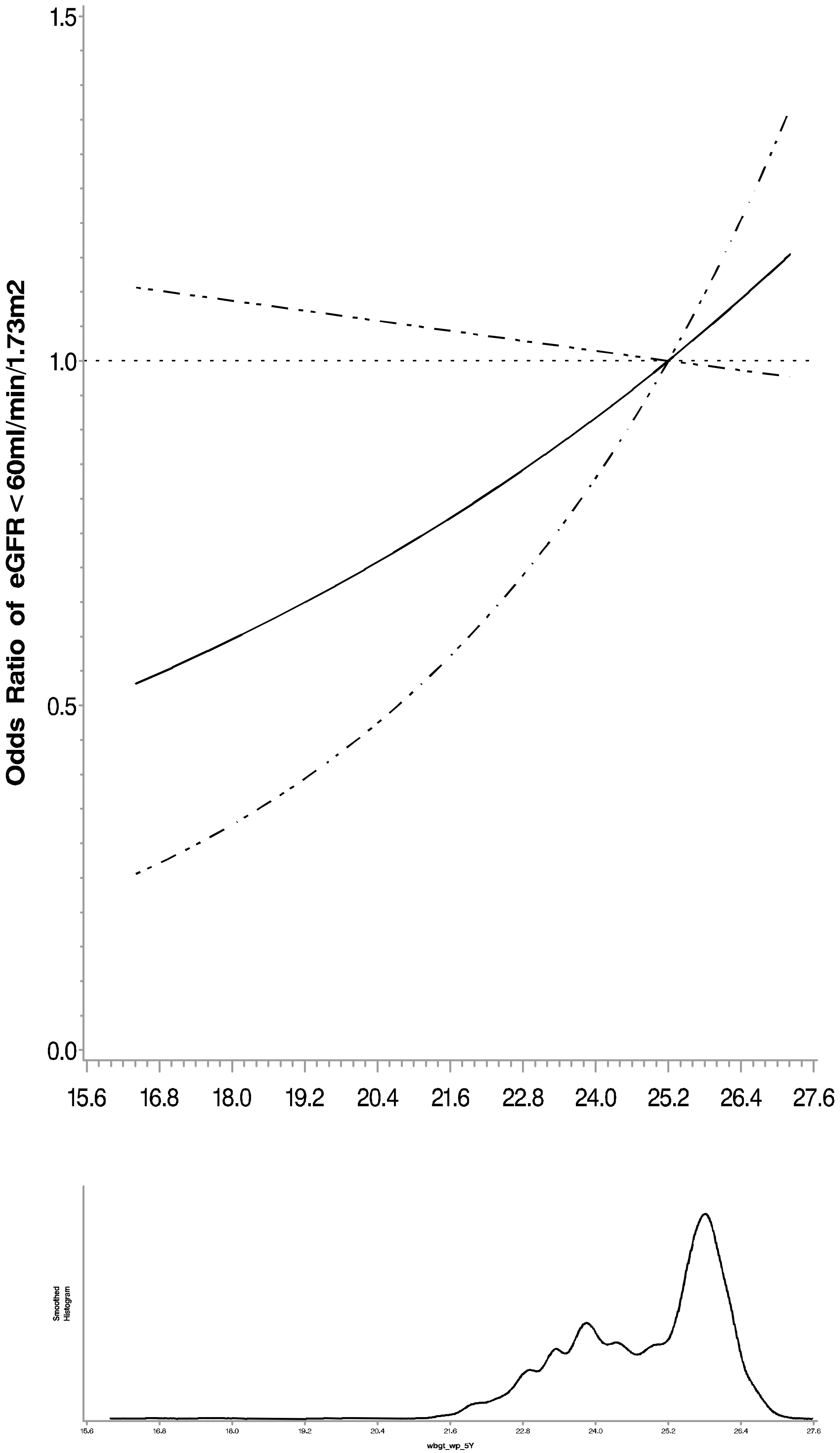

Supplementary Figure 1−6. Restricted cubic spline model of the odds ratios of eGFR<60ml/min/1.73m2 with 5Y WBGT(Work) (Male)

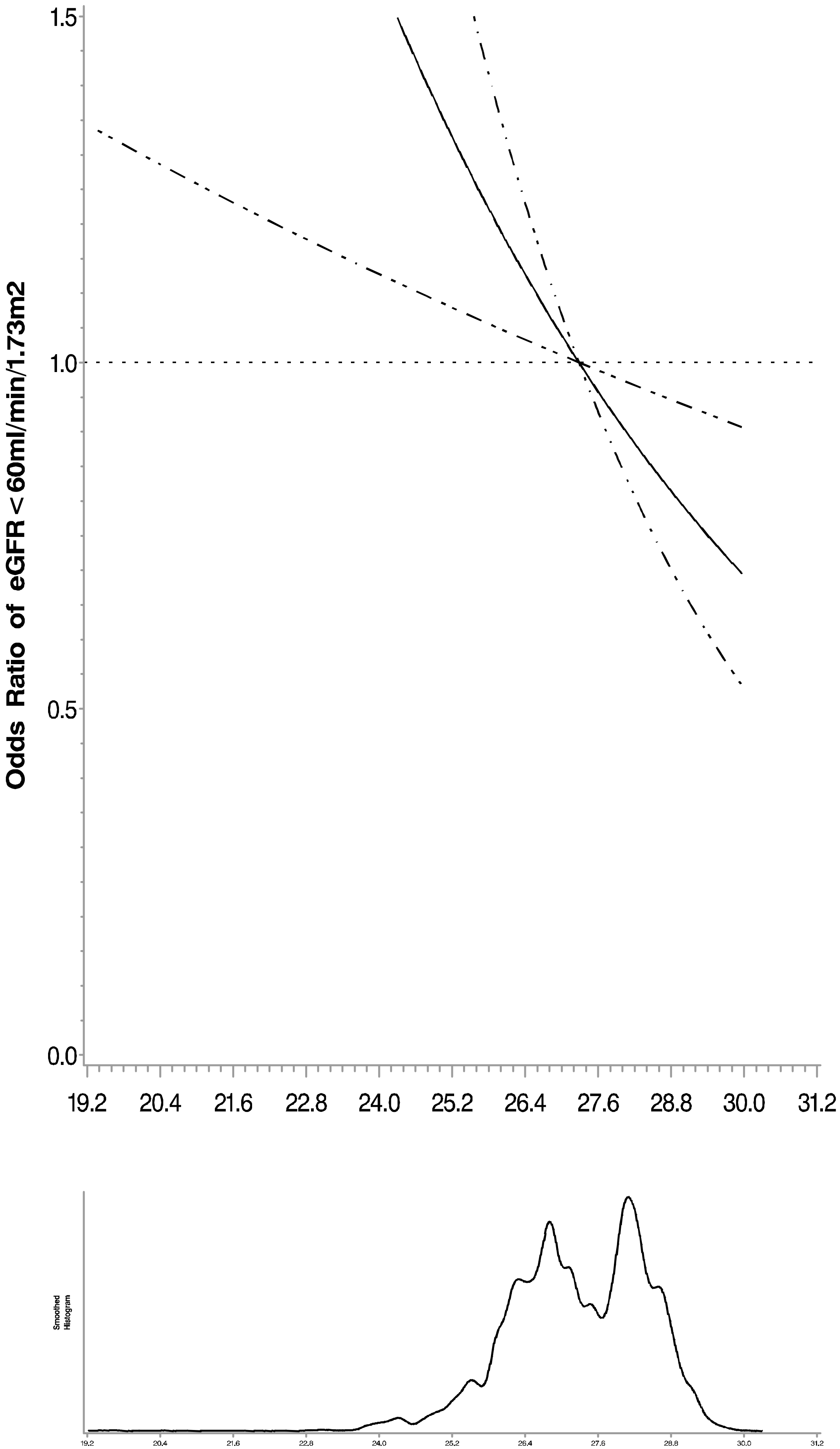

Supplementary Figure 2−1. Restricted cubic spline model of the odds ratios of eGFR<60ml/min/1.73m2 with 1Y WBGT(Noon) (Female)

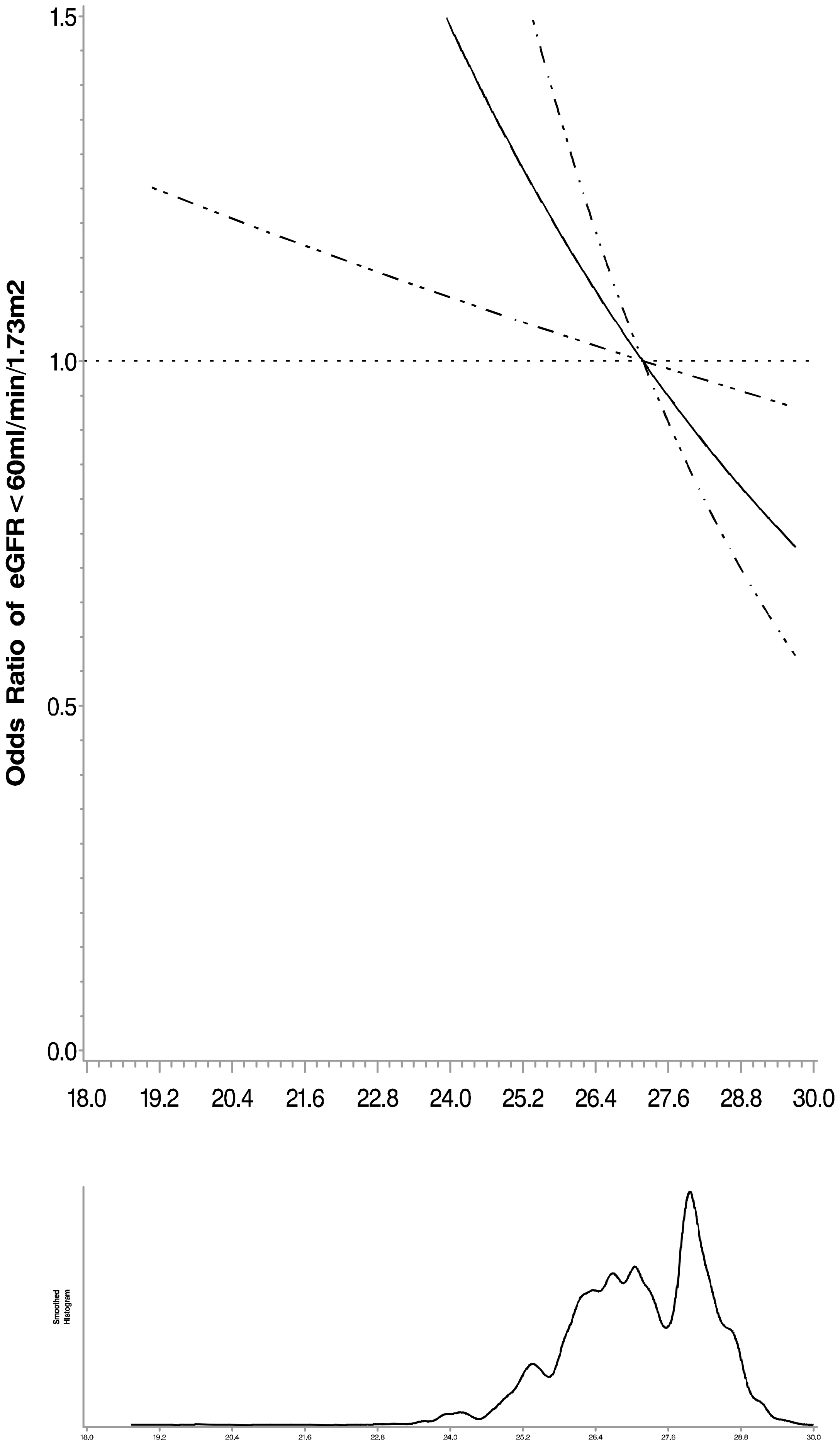

Supplementary Figure 2-2. Restricted cubic spline model of the odds ratios of eGFR<60ml/min/1.73m2 with 3Y WBGT(Noon) (Female)

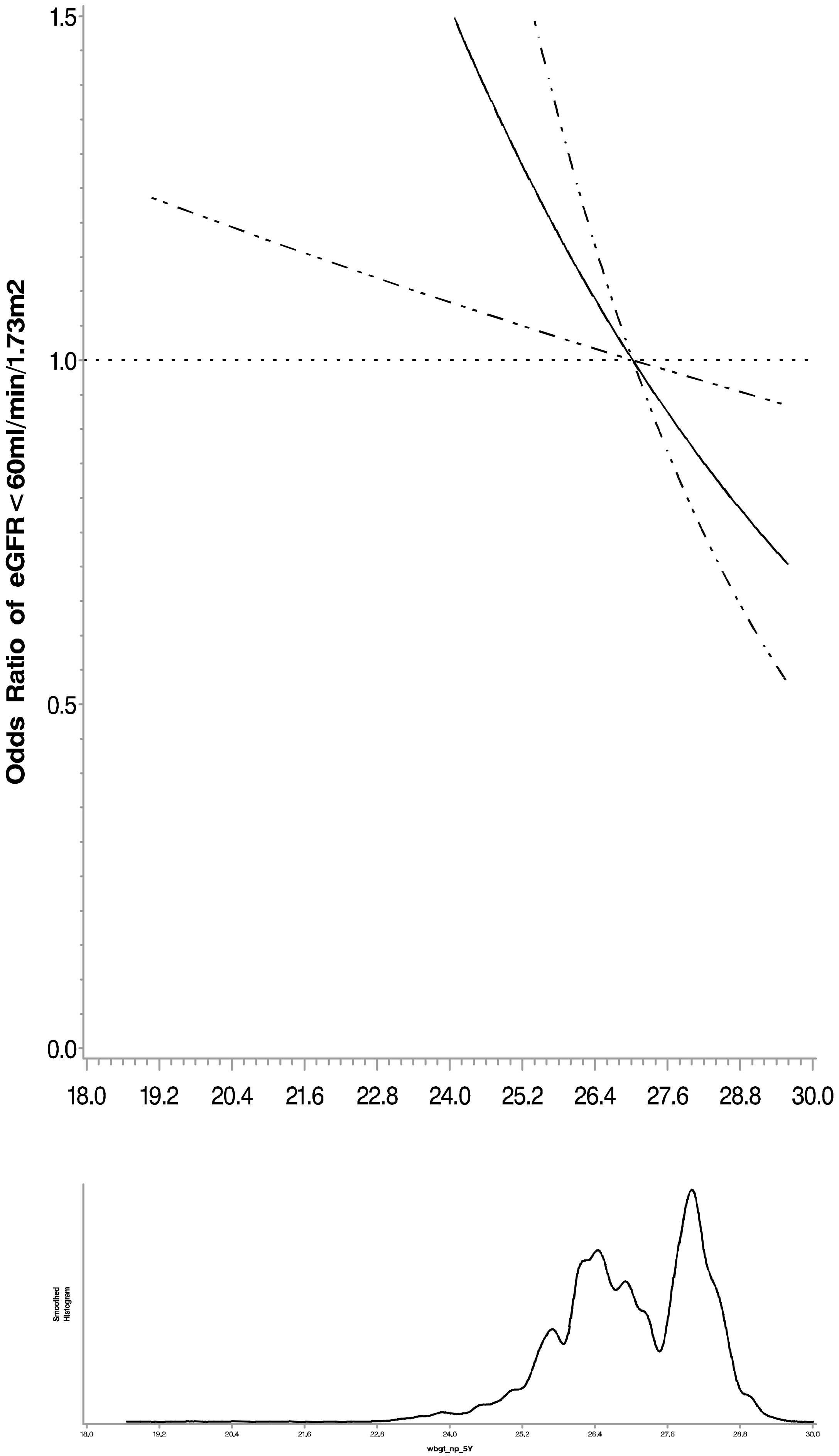

Supplementary Figure 2-3. Restricted cubic spline model of the odds ratios of eGFR<60ml/min/1.73m2 with 5Y WBGT(Noon) (Female)

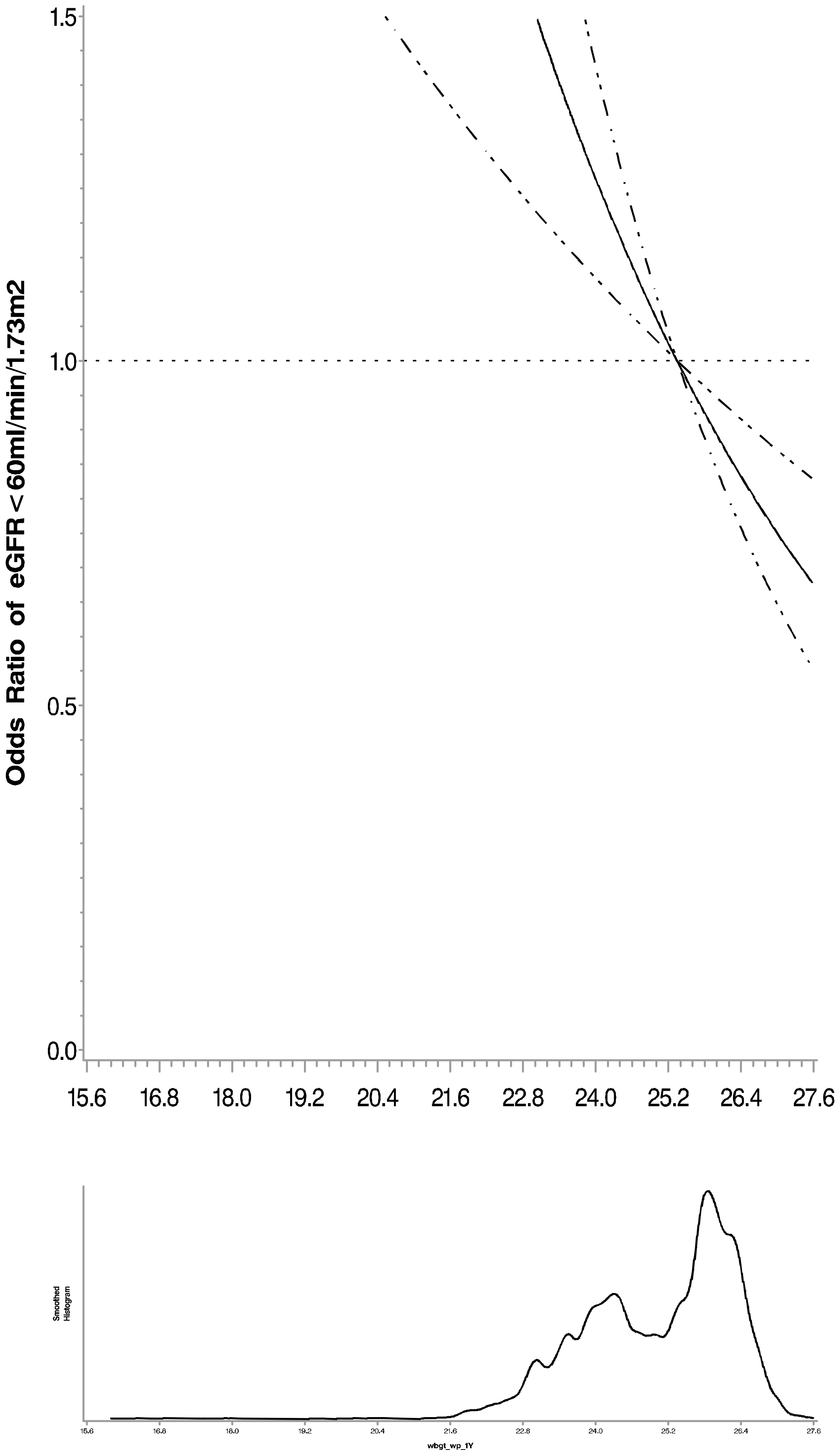

Supplementary Figure 2−4. Restricted cubic spline model of the odds ratios of eGFR<60ml/min/1.73m2 with 1Y WBGT(Work)(Female)

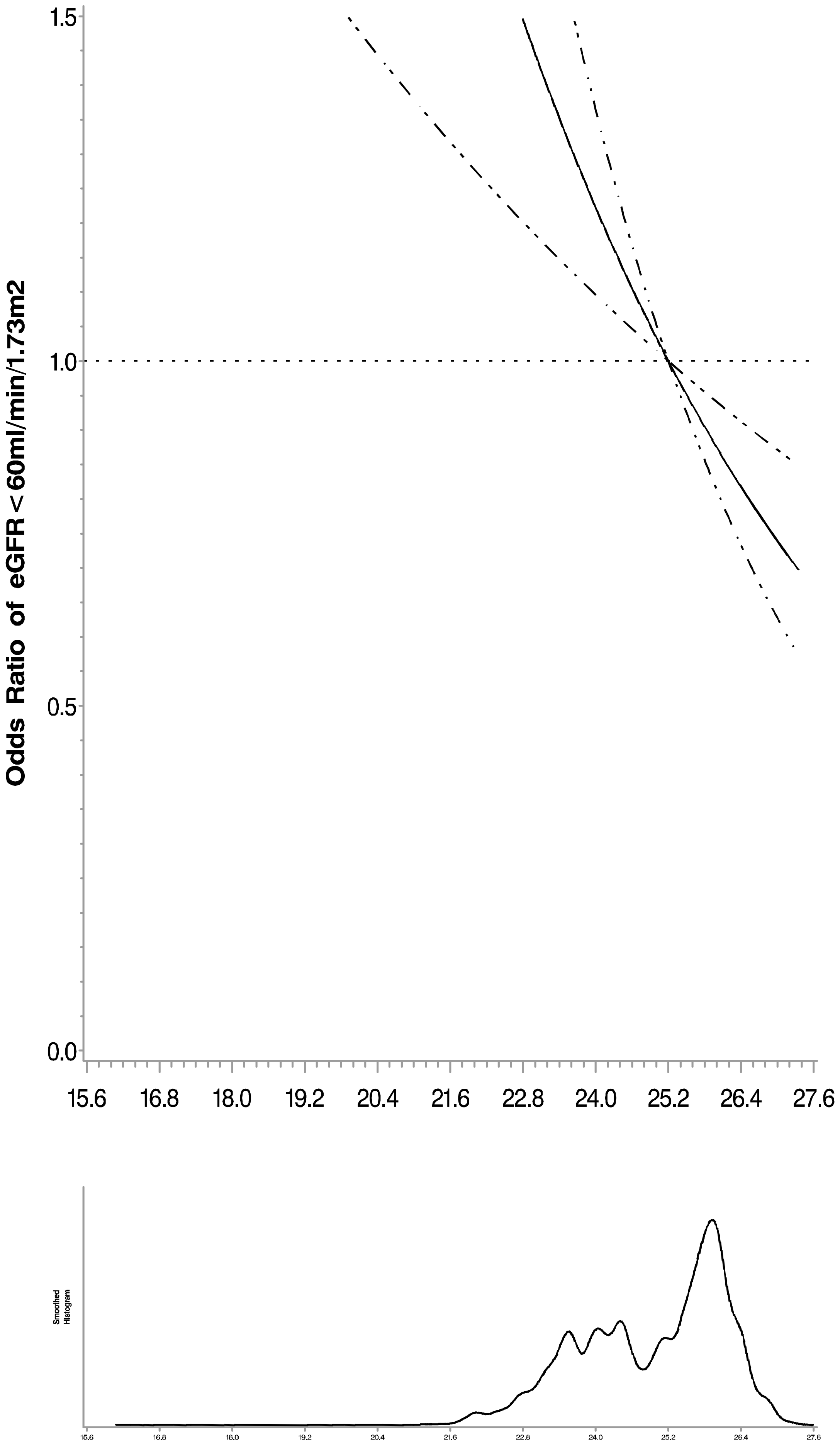

Supplementary Figure 2-5. Restricted cubic spline model of the odds ratios of eGFR<60ml/min/1.73m2 with 3Y WBGT(Work) (Female)

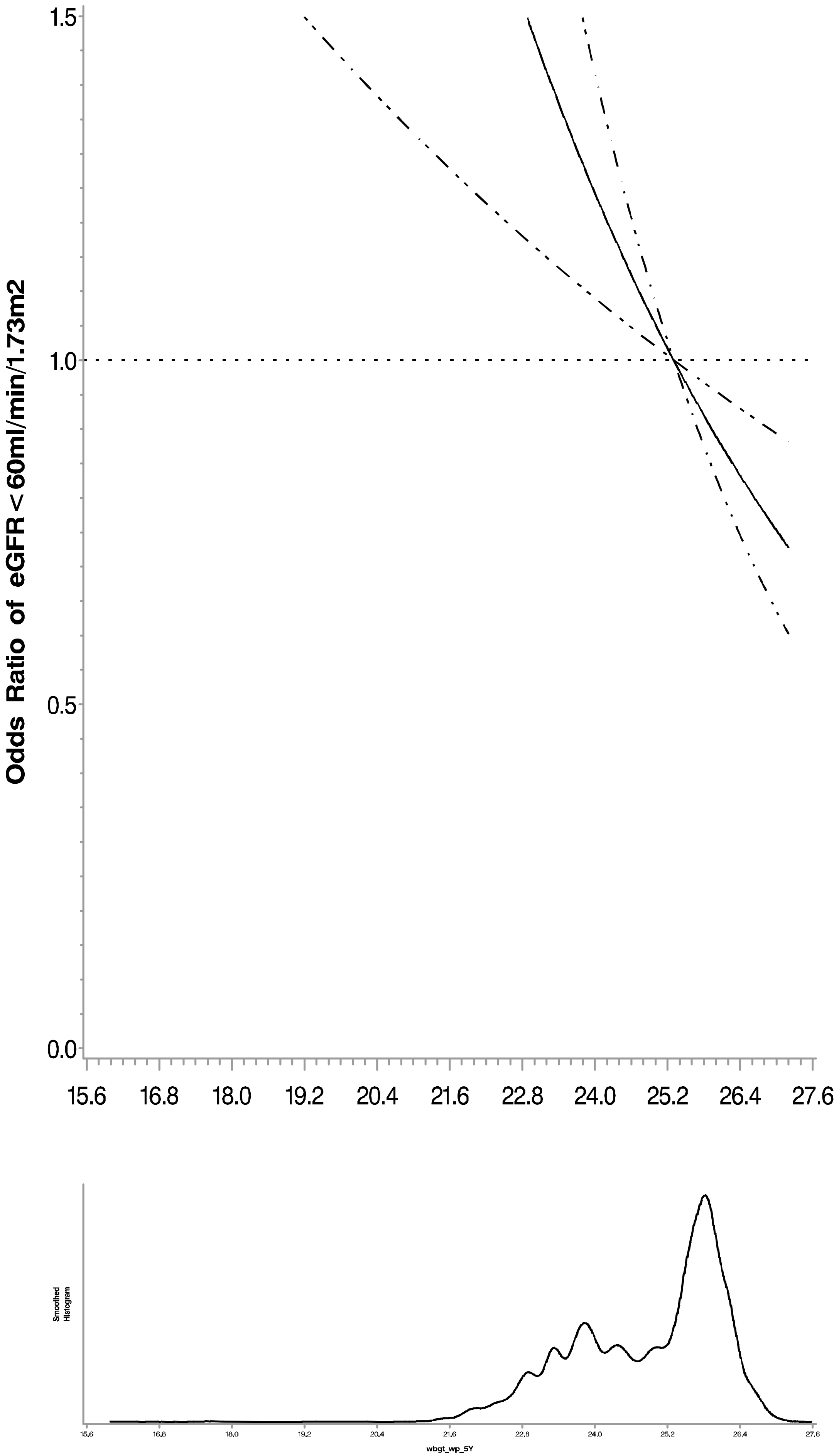

Supplementary Figure 2−6. Restricted cubic spline model of the odds ratios of eGFR<60ml/min/1.73m2 with 5Y WBGT(Work) (Female)
